# Supplementary material for: Fatty Acid Profile and Escherichia coli and Salmonella sp. Load of Wild-Caught Seaweed Fly Fucellia maritima (Haliday, 1838) (Diptera: Anthomyiidae)
Source: Insects. 2024 Feb 28;15(3):163. doi: 10.3390/insects15030163 (PMC10971112; doi:10.3390/insects15030163)
Supplement: Supplementary file 1 [file insects-15-00163-s001.zip › insects-2825047-supplementary.pdf]

*Communication*

# Fatty Acid Profile and *Escherichia coli* and *Salmonella* sp. Load of Wild-Caught Seaweed Fly *Fucellia maritima* (Haliday, 1838) (Diptera: Anthomyiidae)

**Table S1**. Raw data of relative abundance (%) of fatty acids (FAs) present in the adult flies of *Fucellia maritima.* Each Area is one replicate.

| Peaks | Area 1 | Area 2 | Area 3 | Area 4 | Area 5 | Mean | Deviation |
|---|---|---|---|---|---|---|---|
| C12:0 | 0.01 | 0.10 | 0.05 | NA | 0.05 | 0.04 | 0.04 |
| C14:0 | 0.68 | 1.51 | 1.17 | 0.37 | 0.90 | 0.93 | 0.44 |
| C14:1 | 0.26 | 0.39 | 0.18 | 0.08 | 0.25 | 0.23 | 0.11 |
| C15:0 | 0.16 | 0.41 | 0.48 | 0.34 | 0.37 | 0.35 | 0.12 |
| C15:1 | NA | 0.14 | 0.11 | NA | 0.23 | 0.10 | 0.10 |
| C16:0 | 14.62 | 15.61 | 16.89 | 11.82 | 15.45 | 14.88 | 1.89 |
| C16:1 | 31.11 | 36.95 | 31.33 | 33.80 | 41.42 | 34.92 | 4.33 |
| C17:0 | 0.14 | 0.23 | 0.34 | 0.29 | 0.13 | 0.23 | 0.09 |
| C17:1 | 0.65 | 1.26 | 1.21 | 1.42 | 1.69 | 1.25 | 0.38 |
| C18:0 | 2.72 | 2.23 | 2.89 | 2.34 | 1.73 | 2.38 | 0.45 |
| C18:1 | 33.39 | 29.27 | 28.03 | 32.36 | 28.93 | 30.40 | 2.34 |
| C18:2 | 5.21 | 2.66 | 3.72 | 3.73 | 1.71 | 3.40 | 1.31 |
| C18:3 | 2.43 | 2.91 | 5.55 | 5.00 | 1.05 | 3.39 | 1.86 |
| C20:4 | 1.70 | 0.91 | 0.95 | 0.97 | 0.97 | 1.10 | 0.34 |
| C20:5 | 6.67 | 4.99 | 6.87 | 7.14 | 4.66 | 6.07 | 1.15 |
| w3 | 9.10 | 7.91 | 12.41 | 12.14 | 5.70 | 9.45 | 1.23 |
| w6 | 6.91 | 3.57 | 4.67 | 4.70 | 2.68 | 4.51 | 0.91 |
| w6/w3 | 0.76 | 0.45 | 0.38 | 0.39 | 0.47 | 0.49 | 0.14 |

**Table S2.** Raw data of the quantification of phospholipids present in the adult flies of *Fucellia maritima*

| | | Average | ug P | ug lipid | Sum | % |
|---|---|---|---|---|---|---|
| **F. maritima_1** | CL | 0.1476 | 0.2148 | 5.3695 | 59.3485 | 9.0548 |
| | PA | 0.0712 | 0.0446 | 1.1146 | | 1.8796 |
| | PC | 0.2366 | 0.4132 | 10.3294 | | 17.4188 |
| | PE | 0.6922 | 1.4289 | 35.7222 | | 60.2399 |

| | | | | | | |
|---|---|---|---|---|---|---|
| | PI | 0.1140 | 0.1399 | 3.4970 | | 5.8971 |
| | PS | 0.1107 | 0.1326 | 3.3159 | | 5.5917 |
| **F. maritima_2** | CL | 0.1312 | 0.1782 | 4.4555 | 58.3649 | 7.6293 |
| | PA | 0.0741 | 0.0509 | 1.2734 | | 2.1805 |
| | PC | 0.2277 | 0.3933 | 9.8334 | | 16.8380 |
| | PE | 0.6944 | 1.4338 | 35.8449 | | 61.3782 |
| | PI | 0.1125 | 0.1365 | 3.4134 | | 5.8449 |
| | PS | 0.1148 | 0.1418 | 3.5444 | | 6.0691 |
